# The modern-day “Rest Cure”: “The yellow Wallpaper” and underrepresentation in clinical research

**DOI:** 10.1186/s13010-024-00158-8

**Published:** 2024-06-13

**Authors:** Camille Francesca Villar

**Affiliations:** School of Medicine, Baylor College of Medicine, Houston, TX 77030 USA

**Keywords:** Intersectionality, “The Yellow Wallpaper”, Gothic Literature, Clinical Research

## Abstract

Gothic literature—a genre brimming with madness, supernaturalism, and psychological terror—offers innumerable case studies potentially representing how psychiatric patients perceive their treatment from healthcare professionals. Charlotte Perkins Gilman’s famous 1892 short story “The Yellow Wallpaper” offers a poignant example of this through its fictional narrator, a diarist many interpret to be suffering from postpartum depression. The fiction here does not stray far from reality: Gilman orchestrated her diarist’s experience to mirror her own, as both real author and fictional character suffocated from a melancholy only made worse by their physicians’ insistence on following the “Rest Cure.” While this instruction to cease all work and activity was a prevalent depression treatment at the time, Gilman, through “The Yellow Wallpaper,” reveals how the intervention ultimately harmed more than helped because it overlooked her—and, by extension, her fictional diarist’s— unique needs and identities. Today, while the ineffective Rest Cure no longer exists, applying observations from “The Yellow Wallpaper” to clinical research calls attention to underrepresentation in treatment development, a costly problem that could be mitigated by mindful incorporation of intersectionality theory into study designs.

## Background

Narrative literature offers innumerable rich character studies that invite readers to understand the perspectives of others. These stories have relevance in real-world contexts—such as healthcare and research— that depend upon empathetic interactions. In particular, gothic works—brimming with madness, supernaturalism, and psychological terror— are rife with themes ideal for exploring how literature can inform our understanding of psychiatric patients and their treatment. An occult offshoot of realist fiction, many attribute the gothic genre as originating with Horace Walpole’s *The Castle of Otranto* (1764) [1], a novel known for its curious mix of horror and the supernatural in the form of ghosts, murder, foreboding castle imagery, and ominous visions [2]. Over time, authors such as Ann Radcliffe, Mary Shelley, Charles Brockden Brown, Edgar Allan Poe, and Charlotte Perkins Gilman adapted elements from their gothic predecessors’ examples to publish some of today’s most memorable, well-recognized gothic works [1]. While the gothic genre spans poetry, drama, and media beyond that which is strictly literary, this commentary focuses on narrative fiction, with prose that has the potential to elaborate on both character interiority and narrative events. When read with a focus on these character experiences and perceptions, gothic literature provides an accessible, broad-ranging supply of case studies potentially representing how psychiatric patients perceive the world as well as how others perceive them. Reader responses evoked by such literature can, in turn, serve as potent motivation to improve current research practices in treatment development.

Charlotte Perkins Gilman’s classic 1892 gothic short story “The Yellow Wallpaper,” a haunting diaristic record of a young woman’s postpartum descent into madness [3], serves as a particularly noteworthy psychiatric commentary due to its intentional parallels to Gilman’s own life: while historians today tout Gilman as a strong voice for women’s rights, for taking “male forms—in literature and life—and redesign[ing] them to fit all humanity” [4, p. xxi], her path to becoming a powerful feminist involved suffocating struggles with mental health, ineffective prescriptions, and misogyny. Soon after marriage and childbirth, the author (as well as the fictional diarist in her short story) journaled that she suffered from “nervous prostration” ([5], as cited in [6], p. 117). In 1887, Gilman ultimately sought treatment from Dr. Weir Mitchell [6], a physician she explicitly named in “The Yellow Wallpaper” a few years later [3]. Weir Mitchell prescribed the author his now-controversial “Rest Cure,” a treatment consisting of “extreme rest” [6, p. 127] and “total seclusion” [6, p. 127]. Mitchell’s development of the Rest Cure was, in part, informed by the physician’s own opposition to “women of independent spirit” [6, p. 128]; in fact, he believed that “many of the problems he saw in his female patients came from their failure to appreciate” [6, p. 128] the differences between the sexes. In line with this “deeply traditional approach to women” [6, p. 128], Weir Mitchell discharged Gilman from his care with the following prescription, as transcribed in the author’s autobiography:

“Live as domestic a life as possible. Have your child with you all the time. …Lie down an hour after each meal. Have but two hours’ intellectual life a day. And never touch pen, brush, or pencil as long as you live.” [7, p. 96]

In response, Gilman “went home, followed those directions rigidly for months, and came perilously near to losing [her] mind. The mental agony grew so unbearable” [7, p. 96]. While the main oppressive physician in “The Yellow Wallpaper” is the diarist’s husband John, Weir Mitchell is mentioned as an even more undesirable alternative that the diarist wishes to avoid at all costs, since “he is just like John and my brother [another physician], only more so!” [3, p. 6].

A little over a decade following the initial publication of “The Yellow Wallpaper,” Gilman reflected on her experiences and intentions when writing the piece: after three months of Rest Cure placed her “near the borderline of utter mental ruin” [8], she “cast the noted specialist’s advice to the winds and went to work again” [8]. Through her work, she recovered “some measure of power” [8] and authored “The Yellow Wallpaper” as a gothic fiction featuring close parallels to her own experiences. The author relished overcoming both her mental illness and Weir Mitchell’s treatment by triumphantly sending the published story “to the physician who so nearly drove [her] mad” [8].

The real-world translatability of psychiatric illness in “The Yellow Wallpaper,” therefore, comes across especially clearly: the narrator (diarist) in the story finds herself at that same “borderline of utter mental ruin” where Gilman herself felt stranded until she took matters into her own hands and rejected her treatment, the respected Rest Cure. While the relevance of “The Yellow Wallpaper” to the study of medicine and mental health has previously been recognized for its insight into the devastating potential of postnatal psychosis [9], the consequences of social isolation in the context of the COVID-19 pandemic [10], or the constraints of male medical authority [11], it can offer further value as a case study in the disastrous effects of bias in treatment development and application.

Although the ineffective Rest Cure no longer exists, physicians’ dismissal of the diarist’s unique needs and identity mirrors today’s paucity of intersectionality considerations in clinical research, with “intersectionality” referring to the idea that one’s various social identities such as race, gender, socioeconomic status, and disability contribute to compounded systems of oppression that uniquely shape individuals’ experiences, opportunities, and access to resources (including and especially in a healthcare context) [12]. Here, I define “The Yellow Wallpaper” diarist’s identity in the context of intersectionality; using this lens, I offer a reading of the short story in which the problematic Rest Cure—a product of physicians’ ignorance of the diarist’s multifaceted identity—invites questions regarding the circumstances of its genesis in the first place: what were the flaws in the Rest Cure’s development that ultimately led to its failure when applied to patients such as the diarist? These insights call attention to remaining gaps in healthcare and research, heavily underscoring the need for increased focus on intersectionality in the generation of health knowledge through clinical research.

## The diarist: a nameless identity

The illness identity defined by a clinical diagnosis never exists in a void; rather, diagnoses must be situated at intersectional crossroads of patients’ identities. The diarist in “The Yellow Wallpaper,” though nameless, is not anonymous; rather, she exhibits extensive individuality through the myriad identities that contribute to her unique social situation and perspective. Firstly, she is a woman and a wife in the patriarchal society of 19th -century America,^1^ pressured by social, medical, and familial norms to follow her husband’s direction, as the men in the diarist’s life—primarily, her husband and brother—are physicians of “high standing” [3, p. 1]. Taken with the fact that John is “one’s own husband” [3, p. 1], the diarist sees herself as having no choice as to what she does with herself: “what is one to do?” [3, p. 1], presumably because she believes society— “friends and relatives” [3, p. 1]—will side with the opinions of the male physicians.

Secondly, she holds a maternal identity with self-appointed responsibilities within her family, as she wishes to serve as a nurturing mother to her recently born child.^2^ In regard to her own sense of self, she also identifies as a writer, as evidenced by her commitment to authoring the diary despite medical advice to the contrary.^3^ She struggles with a mental health identity consistent with postpartum depression with psychotic features, expressed through the following postpartum symptoms: “I cry at nothing, and cry most of the time” [3, p. 6]; she laments her slothfulness, reprimanding herself because “Half the time now I am awfully lazy, and lie down ever so much” [3, p. 7]; she hallucinates a woman imprisoned within her room’s wallpaper: “At night in any kind of light… it becomes bars! The outside pattern I mean, and the woman behind it as plain as can be” [3, p. 10].

Despite her struggles with mental health, she still is publicly identifiable as a reasonably wealthy woman (inferred from the “mansion”^4^ of the story’s setting and her husband’s professional identity as a physician [3, p. 1]), a position of relative privilege that potentially contrasts with her gender and mental subjugation. In other words, the diarist captures the complicated nature of intersectionality: for example, while her mental condition robs her of autonomy, she still comes from a background that allows her to rest in a mansion and receive regular—albeit biased and dismissive— healthcare from her husband. Moreover, we can appreciate that while her opportunities are limited due to her gender, they do not appear to be similarly limited by her race.^5^

Overall, while some of the diarist’s aforementioned identities—such as socioeconomic status and race— work to her advantage, other identities (in line with newer interpretations of “intersectionality” that consider qualities beyond that of gender and race, such as disability [17]) detrimentally clash and compound. For example, her mental impairment augments her gender to maximally justify the obeisance her husband demands. At another intersection, the impact of her condition as a woman with postpartum depression is rendered more severe due to the handicap it places on her from fulfilling the responsibilities she assumes as a mother as well as from completing personal projects—such as writing in the diary more often^6^— as a writer.

The diarist’s various roles, self-perceptions, perceived societal expectations, and intrinsic circumstances intersect at numerous distinct loci to produce her specific intersectional identity, the unique background that gives further context to her presentation as a psychiatric patient. This does not even begin to cover the identities the diarist develops over the course of her illness journey, in which her conviction in her own experiences (“I am sick!” [3, p. 1]) wars with the minimizing label given by her physician husband (“there is really nothing the matter with one but temporary nervous depression” [3, p. 1]): her self-perceived illness identity differs vastly from that attributed to her by others, and this discrepancy, on top of the complications and issues tied to her other intersecting identities, contributes to the frustration she feels as a patient.^7^

## The “Rest Cure” of “The yellow Wallpaper”: dismissal and unempathetic treatment

While readers can parse numerous layers of the diarist’s identity as an upper-middle-class white woman, mother, wife, writer, and person with depression, the physicians of the story (namely, her husband John) never do the same, and in turn they neglect to consider how these identities mediate the effects of the generally accepted treatment.

The primary interactions in “The Yellow Wallpaper” are between patient and physician (the diarist and her husband); the story establishes the lopsided power dynamic of these interactions early on:

John is a physician, and perhaps— (I would not say it to a living soul, of course, but this is dead paper and a great relief to my mind)— perhaps that is one reason I do not get well faster.

You see he does not believe I am sick!

And what can one do? [3, p. 1]

This quote, deliberately situated early in the story, immediately associates John with his profession as “a physician.” Rather than elaborating on their relationship as husband and wife, Gilman positions the diarist as a patient and her husband as a (presumably) knowledgeable medical professional. Immediately, then, she establishes a power dynamic: the diarist thinks herself too foolish— or John too knowledgeable— to feel worthy enough to voice her own opinion on her condition (“I would not say it to a living soul”), and she feels powerless to oppose his minimization of her symptoms (he “does not believe” she is sick, that she needs further treatment than rest, and the diarist’s immediate response is to defer to his judgment—“And what can one do?”). As the story unfolds from the perspective of the diarist, readers become intimately acquainted with the subjugated side of this relationship. Consequently, they are positioned to clearly understand the diarist’s struggles, and, in turn, experience empathy and concern greater than that exhibited by any other character in the story.

As “The Yellow Wallpaper” progresses, the power dynamic remains evident: the diarist’s assessments of herself and her inclination for certain treatments continue to conflict with John’s own diagnoses and prescriptions. The following excerpt features this tiring, helpless opposition between the diarist and John, between suffering patient and supposedly omniscient physician:

If a physician of high standing, and one’s own husband, assures friends and relatives that there is really nothing the matter with one but temporary nervous depression—a slight hysterical tendency—what is one to do?

My brother is also a physician, and also of high standing, and he says the same thing.

So I take phosphates or phosphites—whichever it is, and tonics, and journeys, and air, and exercise, and am absolutely forbidden to ‘work’ until I am well again.

Personally, I disagree with their ideas.

Personally, I believe that congenial work, with excitement and change, would do me good.

But what is one to do?

I did write for a while in spite of them; but it does exhaust me a good deal—having to be so sly about it, or else meet with heavy opposition.

I sometimes fancy that in my condition if I had less opposition and more society and stimulus—but John says the very worst thing I can do is to think about my condition, and I confess it always makes me feel bad. [3, pp. 1–2]

The diarist feels her arguments with “a physician of high standing” are discouragingly unwinnable. Thus, she obediently takes her medications, simultaneously belittling herself and emphasizing John’s complete control over her treatment plan by commenting how she did not even know “whichever it is,” “phosphates or phosphites.” She takes John’s instructions as commands, seeing herself as “absolutely forbidden” to engage in whatever he prohibits.

Despite her outward obeisance, she admits she “disagree[s] with their ideas.” Contrary to John’s instructions, she believes “that congenial work, with excitement and change, would do me good.” She even wrote in her diary “in spite of them,” imagining her ideal treatment with “less opposition and more society and stimulus.” In these moments, she stood on the brink of achieving the same positive outcome Gilman herself found upon attempting “to work again—work, the normal life of every human being; work, in which is joy and growth and service” [8]; for Gilman, continuing to work meant “ultimately recovering some measure of power” [8].

Notwithstanding these glimmers of the diarist’s autonomy, readers remain powerless witnesses of her divergence from Gilman as she constantly, unfailingly retreats back into her role as a helpless, ignorant patient. Failing to reconcile conflicting treatment ideas, she ultimately convinces herself of John’s credibility: she reasons that he was right in saying “the very worst thing I can do is to think about my condition,” because she admits “it always makes me feel bad.” The physician continually silences—and, in turn, neglects— his patient’s perspective on how the Rest Cure handicaps her sense of self. The desperation in her repeated mantra “what is one to do?”, while written ostensibly in private to her fictional self, amplifies into a call for help in the minds of readers as the dissonance between patient and treatment crescendos.

Gilman leverages this friction, this constant opposition between the diarist and the “physician of high standing,” to orchestrate an unconventionally triumphant ending. After John returns from a trip, he finds that his wife had succumbed to complete psychosis:

He stopped short by the door.

What is the matter?” he cried. “For God’s sake, what are you doing!

I kept on creeping just the same, but I looked at him over my shoulder.

I’ve got out at last,” said I, “in spite of you and Jane? And I’ve pulled off most of the paper, so you can’t put me back!

Now why should that man have fainted? But he did, and right across my path by the wall, so that I had to creep over him every time! [3, p. 15]

Preceding John’s discovery of his wife in this state, the diarist had spent the day lost among her hallucinations of the woman trapped in the yellow wallpaper. Ultimately, her warped perception of reality evolved such that the diarist imagined herself as the woman imprisoned within the paper. John returns, and the diarist ignores his presence as she crawls throughout the hallway— “I kept on creeping just the same.” She explains that, “in spite of” John, she “got out at last,” and she defiantly cries to him, “you can’t put me back!” Ultimately, the power dynamic is finally reversed, with John fainted on the ground and the diarist figuratively and literally above him, crawling “over him.”

Here, a full spiral into psychosis is a preferable, more triumphant fate over constantly being at odds with John’s treatment. John had dismissed his wife’s condition— “there is really nothing the matter”— and gave ineffective treatment (the “absolute” forbiddance to work, which uncoincidentally mirrors Weir Mitchell’s Rest Cure) up until this point. Only at the end of the story does the diarist finally become free: she “got out at last” from the suffocating prescriptions and lack of empathy offered by John. One possible interpretation of this ambiguous ending, therefore, is the suggestion that dissembling this helpless patient-physician power dynamic that enforced inappropriate, harmful treatment by any means necessary (even if that requires giving in to madness and hallucinations) is the key to a mentally sick woman’s freedom.^8^

For the entirety of the story, the diarist was on the brink of recovery, of engaging in “congenial work, with excitement and change” just like Gilman herself did. However, John’s constant belittlement, dismissal of the seriousness of her condition, and ignorance of his wife’s feedback on her mental status^9^ contributed to his maintenance of unfounded confidence in treatments that ultimately kept the diarist trapped behind the bars of her postpartum depression.

John blindly backed the Rest Cure as the definitive treatment for his wife, and his use of authoritative condescension in place of empathetic dialogue further demonstrated his confidence in himself and his prescription. He did not recognize that the Rest Cure directly conflicted with her perception of herself as a writer, for example, or her desire to act as an involved mother for her newborn child; he did not recognize that his belittlement shifted the already-skewed husband-wife power dynamic further into his favor, effectively silencing any protest the diarist may have otherwise made. Rather, John trusted a standard treatment that only accelerated his wife’s succumbing to madness, a treatment strikingly similar to the Rest Cure of dubious scientific backing and flimsy, assumed generalizability devised by Weir Mitchell—who is “just like John… only more so!”

As a whole, “The Yellow Wallpaper” forges a connection between the diarist and readers in which readers gain unique insight into the harmful opposition between identity-driven priorities and conventional treatment. The unsettling means the diarist subconsciously resorts to in order to finally gain freedom at the end of the story leaves readers with a dissatisfying resolution to her struggles, prompting the question: how could this have been avoided?

## The “Rest Cure” of today: the need for intersectionality in clinical research

Medicine and physician-patient relationships have noticeably improved in the century since the publication of “The Yellow Wallpaper”; overall, marginalized identities in healthcare have garnered many advocates, and treatments with little-to-no scientific basis, such as the Rest Cure, have been put out of practice. Nonetheless, the message communicated in this short story provides valuable insights for modern-day clinical researchers.

In both the real and fictional cases of Gilman and the diarist, the physicians prescribed treatment that was generally accepted at the time for women with the general symptoms described. John and the diarist’s brother both provided the same advice consisting of Rest Cure elements as well as pharmacologic interventions (“phosphates or phosphites—whichever it is, and tonics”), which we could only presume from their “high standing” is a treatment regimen aligning with the standard of care for women of “temporary nervous depression.” Weir Mitchell, understood by Gilman to be “At that time the greatest nerve specialist in the country” [7, p. 95], designed his Rest Cure after he restored “Mrs. B,” a married mother of three presenting with sudden weight loss and amenorrhea [6, p. 125]. With seclusion, bed rest, and frequent feeding, she recovered; this led Weir Mitchell to conclude “he had devised a therapy for a disease he could not identify and would not name” [6, p. 127]. The treatment had somewhat replicable results, as “for some women, the combination broke through the cycle of insomnia, self-starvation, and drugging that had been destroying them” [6, p. 127]. Mitchell publicized his Rest Cure in his 1877 publication, *Fat and Blood: And How to Make Them*, which “brought him a broad popular audience, and underlay the tremendous growth of his practice” [6, p. 125].

In short, Gilman and the diarist both likely received an acceptable standard of care at the time—a standard of care established by a patriarchal society that had no problem with Weir Mitchell’s “deeply traditional approach to women” and his uncomprehensive method of validating his Rest Cure. Nevertheless, those standard treatments failed: the diarist’s symptoms worsened, and Gilman had to abandon her treatment plan to recover. These failures were not due to the treatment’s lack of backing and approval from educated peers (such as the diarist’s brother, who agreed with John’s treatment plan [3, p. 1]) nor from the medical research community (Weir Mitchell “first presented his new therapy in a lecture to a medical audience” [6, p. 125] and was met with receptiveness from “fellow physicians” [6, p. 125]). In the case of the Rest Cure and presumably in the case of John’s prescriptions, the treatment had been used successfully in the past with other patients, or, at the very least, commonly held belief in its merits resulted in its nationwide endorsement amongst physicians.

The problem was not with whether the Rest Cure was seen as an acceptable treatment by the medical community; rather, the problem concerned whether the Rest Cure was fitting for the specific, intersectional patient population Gilman and the diarist occupied. The main barrier to the diarist’s effective treatment was the fact that the conceited “physician of high standing” never allowed his wife to voice her experience of her mental illness nor of his treatments. Existing as an upper-middle-class woman, mother, and writer with postpartum depression is not a condition that a physician could completely understand without properly eliciting that perspective; the diarist experienced a mental illness so specific to her, yet John never once asked her to communicate her viewpoint. Considering the situation from the level of clinical research for treatment development magnifies this issue: Weir Mitchell’s evidence for his Rest Cure mainly consisted of women reportedly cured of their “cycle of insomnia, self-starvation, and drugging.” Women with these general symptoms comprise an ill-defined patient population, and the treatment was published with equally vague indications (the therapy was “for a disease he could not identify and would not name”). Much like how John never explored the nuances of his wife’s illness and treatment experience, the development of the Rest Cure itself appears to have been characterized by that same close-mindedness: the diversity of identities—illness, occupational, and otherwise— in the Rest Cure’s study population were not acknowledged, and therefore differential outcomes based on intersectionality were not identified. Had a more diverse group of patients been studied—and their diversity appreciated in the interpretation of results— the outcome of applying the Rest Cure to a writer, a patient with postpartum depression, or someone experiencing psychosis would have been better understood.

To readers of “The Yellow Wallpaper,” one physician’s lack of empathy led to the neglect of one patient’s intersectional identity, ultimately leading to an adverse outcome. While the short story offers this singular example, the true number of women similarly victimized by the Rest Cure is likely vast yet ultimately unknowable. Lack of empathy certainly poses a problem on the level of John, but it can be addressed—to much greater effect—on the level of the development of treatment in the first place: the level of clinical research. Indeed, Weir Mitchell’s Rest Cure originated, in part, from his “deeply traditional approach to women”; its design was predicated on prejudice that stunted his ability to see the diversity among his patients. What if the effects of the Rest Cure were studied in more patient populations, spanning and recognizing multifarious combinations of illness identity, disability status, occupation? Would different outcomes have been identified based on these intersectional identities? Likely so, and those findings would have swayed the standard of care for these populations and, in turn, John’s prescription for his wife.

In this way, John’s lack of awareness of his individual patient’s intersectionality in “The Yellow Wallpaper” reflects the lack of population-level intersectional considerations in the Rest Cure’s development. Hypothetically expanding beyond the diarist’s intersectional locus, there was even potential for further—yet historically impossible—improvement of the treatment: studying the Rest Cure across socioeconomic class, gender, and race could have vastly enhanced understanding of its effects and indications, had it been offered to such populations in the first place. Realistically, this diversity in research would not have been possible in Gilman’s or the diarist’s lifetime, and the Rest Cure has no place in today’s medical landscape; however, this takeaway still holds immense value in the realm of clinical research of modern-day treatments: treatments need to be devised, investigated, and approved with patients’ multifaceted, intersectional identities in mind. The National Academies of Sciences, Engineering, and Medicine recently brought to attention the tendency of clinical trials to be less inclusive of historically underrepresented groups such as “non-Hispanic Black men, Hispanic/Latinx men, non-Hispanic white women, non-Hispanic Black women, and Hispanic/Latinx women” [15]. This lack of representation incurs costs of “hundreds of billions of dollars” [15] due to lack of access to effective medical interventions, failure to recognize socially mediated health disparities in underrepresented populations, and overall social costs of decreased life-expectancy, contribution to labor force, and disability-free life [15]. To disastrously costly effect, minority populations lack adequate treatments because of their omittance from the research developing those treatments. This exclusion results in numerous barriers, ranging from minority demographics not being approved for treatment to standard treatments not considering intersectional identities, the social determinants of health tied to them, or both; this latter observation especially echoes the diarist’s struggle for having her intersectionality recognized in “The Yellow Wallpaper.” Our modern-day Rest Cure, therefore, is the devastating lack of relevancy of current health research to underrepresented groups that could not participate in such research.

The above report delineates populations based on the intersection of racial, ethnic, and gender identities, implicitly acknowledging the importance of intersectionality, of defining demographic groups in a more complex manner than the basis of a single identity, in clinical research. Similarly, observations in specialties spanning from surgery [16] to psychiatry [17] point out how the body of knowledge on diagnostics, outcomes, and interventions advances with little consideration to marginalized populations. Current research largely lacks consideration of intersectionality in that it does not adequately explore the impact of compounded marginalized identities on healthcare outcomes [16], and current and developing mental illness models fail to recognize the social norms and pressure resulting from patients’ perceived identities [17]. The diarist in “The Yellow Wallpaper” figures as an exemplar of such a patient with unique identities and priorities: she came for treatment as a dignified upper-middle-class woman, who was also a psychiatric patient perceiving disturbing symptoms she wished to (but could not) express to her husband, with priorities to work as both a mother and writer. She suffered from a treatment developed on a broadly defined psychiatric illness model (her “temporary nervous depression”) with no consideration for differential outcomes based on intersectionality.

Starkly contrasting with the impersonal objectivity of today’s discussion of intersectionality in clinical research, Gilman’s 19th -century short story imbues modern reports’ calls to action with alarming, visceral imperative in a way only the unrestrained form of literature can. “The Yellow Wallpaper”—and the autobiographical experiences that inspired it—garners far-reaching empathy that compels readers to ask how the diarist’s experience could have been improved, how her suffering could have been mitigated at the level of her treatment’s development.

While the need to recognize patient populations at a level even remotely near the nuance with which we understand the diarist in “The Yellow Wallpaper” in clinical research is prominently evident,^10^ the question of how to address this gap remains. For future health research to incorporate intersectionality theory into their methodologies, clinical and public health researchers must first agree upon quantitative models and standardized processing of different intersectional data points. Challenges that collectively need to be addressed in the transition to making intersectionality more pervasive in healthcare research include clarifying confusing statistical terminology with regard to intersectionality, determining which parameters best assess discrimination, and coming to a consensus on how to quantitatively model interactions of different identities using appropriate scales and regression models [19]. While these challenges pose a daunting barrier to timely standardization of quantitative research incorporating intersectionality, studies for the time being may alternatively address underrepresentation in research by using current models to focus on marginalized populations [15] or, in the case of psychiatry, incorporating intersectional frameworks into research question formulation and study design, genetic counseling, and patient-level treatment considerations [17].

## Conclusion

Healthcare is a field comprised of patients, physicians, and researchers of innumerable backgrounds; thus, it is a field that could always benefit from the empathy elicited by realistic stories or retellings of patient experiences that some may not otherwise share. Literature rooted in real-world thoughts, feelings, and experiences (such as how the discrimination Gilman herself faced mirrors the discrimination faced by the diarist), therefore, has especially invaluable potential as a lens for elucidating issues in healthcare. Specifically, when reading the diarist as an exemplar of the ignorance of patients’ intersectionality when considering and researching treatments, “The Yellow Wallpaper” persists as a call for modern-day clinical researchers to appreciate the complex differential interactions between interventions and multifaceted identities.

## Data Availability

Not applicable.

## Footnotes

1 To give further context to the setting of the story, “The Yellow Wallpaper” was originally published in the late 19th century. For more on the patriarchal backdrop of this short story, see Suess (2003), p. 86 [13].

2 While it takes great effort and makes her “so nervous” [3, p. 4] to take care of her “dear baby” [3, p. 3], she still expresses her desire for the child to live their best life, as she finds it “fortunate Mary is so good with the baby” [3, p. 3], and she takes comfort that “the baby is well and happy, and does not have to occupy this nursery with the horrid wallpaper. …What a fortunate escape! Why, I wouldn’t have a child of mine, an impressionable little thing, live in such a room for worlds” [3, p. 8].

3 She believes “that congenial work, with excitement and change, would do me good” [3, p. 1]. She admits she “did write for a while in spite of [her husband and brother]; but it *does* exhaust me a good deal—having to be so sly about it, or else meet with heavy opposition” [3, p. 2].

4 While she calls herself and John “mere ordinary people” [3, p. 1], they have the resources to stay in “A colonial mansion, a hereditary estate” [3, p. 1]. The diarist utilizes the word “cheaply” [3, p. 1] in the context of this mansion being worth vastly much more than the sum the couple paid to rent it for the summer; regardless, a vacation home of this caliber—being relatively cheap or not—still signals wealth, suggestive of the diarist belonging to the upper-middle class. Additionally, it can be inferred that they hire a nursemaid, Mary, to take care of their child [3, p. 3], which indicates the presence of further disposable income.

5 Here, it is important to note that “intersectionality” was first coined in Black feminist studies by Kimberle Crenshaw, and it was used to describe the oppression experienced by Black women, with gender and race compounding to generate barriers greater than if one possessed either identity alone [14]. Given the origins of the term, a discussion of the diarist in the context of intersectionality warrants acknowledgement that her race is white (as largely implied from her upper-middle-class identity in 19th century America and the partially autobiographical nature of the story). By and large, from a Black feminist perspective, the diarist represents a position of relative privilege that cannot be ignored: she had access to treatment (albeit treatment that exacerbated her condition), while her contemporary Black and less economically privileged mothers faced barriers to receiving treatment to begin with. Therefore, admittedly, intersectionality in “The Yellow Wallpaper” fails to yield insight into the nuances of 19th century treatment of Black women with postpartum depression; notwithstanding, the diarist possesses other identities that render her an acceptable example of the interplay between intersectionality and treatment.

6 At the beginning of her second entry, she explains “We have been here two weeks, and I haven’t felt like writing before, since that first day” [3, p. 3].

7 The author would like to thank an anonymous reviewer for providing this valuable insight.

8 This idea has been explored before, such as Paula A. Treichler (1984) pointing out “The fact that her unflappable husband faints when he finds her establishes the dramatic power of her new freedom” [11, p. 67]. Notably, previous readings such as Treichler’s serve a more feminist, antipatriarchal purpose, whereas the present commentary asserts that the diarist’s descent (or, more aptly, triumphant ascent) into madness symbolizes the diarist breaking free of the ineffective, ill-informed medical treatment forced upon her with no consideration of how her intersectional identity would impact outcomes.

9 John never asks for his wife’s opinion on the treatment, and he even silences her when she tries to express how she feels: when he tells her she is better, the diarist begins to protest, but then she “stopped short, for [John] sat up straight and looked at me with such a stern, reproachful look that I could not say another word” [3, p. 9].

10 While this commentary establishes this need through the modern-day gaps highlighted by the National Academies of Sciences, Engineering and Medicine [15], the detrimental effects on surgical outcomes [16], and the lack of intersectional considerations in the modeling and understanding of psychiatric illness [17], further reading on this imperative can be found in Sabatello et al. (2023), in which the authors argue for the importance of incorporating intersectional considerations in precision medicine research [18].
